# Supplementary material for: Gestational diabetes augments group B Streptococcus infection by disrupting maternal immunity and the vaginal microbiota
Source: Nat Commun. 2024 Feb 3;15:1035. doi: 10.1038/s41467-024-45336-6 (PMC10838280; doi:10.1038/s41467-024-45336-6)
Supplement: Supplementary file 1 — Supplementary Information [file 41467_2024_45336_MOESM1_ESM.pdf]

SUPPLEMENTARY INFORMATION

Gestational diabetes augments group B *Streptococcus* infection by disrupting maternal immunity and the vaginal microbiota

Vicki Mercado-Evans, Marlyd E. Mejia, Jacob J. Zulk, Samantha Ottinger, Zainab A. Hameed, Camille Serchejian, Madelynn G. Marunde, Clare M. Robertson, Mallory B. Ballard, Simone H. Ruano, Natalia Korotkova, Anthony R. Flores, Kathleen A. Pennington, and Kathryn A. Patras

Contents:

Supplementary Figures 1-6

Supplementary Tables 1-3

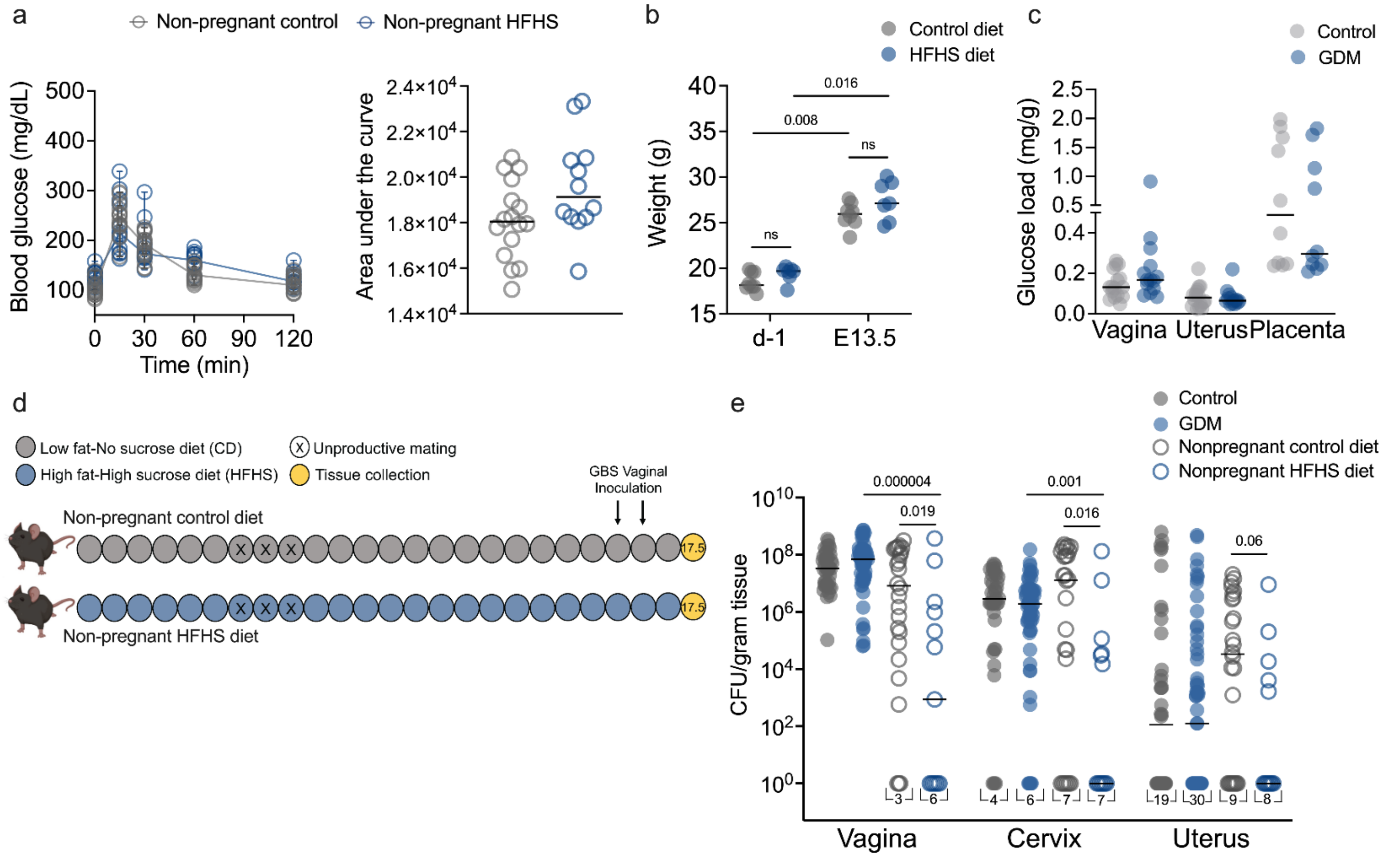

Supplementary Figure 1: Pregnancy and diet differentially impact GBS reproductive tract burdens. a Blood glucose concentration and area under the curve during glucose tolerance test one day before mating. *n* = 16 mice on control diet and. *n* = 12 mice on HFHS diet. **b** Body weights of mice collected one day prior to mating and at E13.5. **c** Tissue glucose levels on E17.5. *n* = 16 vaginal, 16 uterine and 10 placental tissues from GBS challenged control dams on E17.5, and *n* = 14 vaginal, 14 uterine and 10 placental tissues from GBS challenged GDM mice on E17.5. **d** Parallel experimental timeline for non-pregnant mice maintained on either control or high-fat high-sucrose (HFHS) diet followed by vaginal colonization with GBS strain A909 on d14.5 and 15.5 and sacrifice on d17.5 to assess GBS dissemination. **e** GBS burden throughout the reproductive tract where points represent individual mouse samples and lines indicate median CFU per gram of tissue. Mouse image (d) created with BioRender.com. *n* = 39 pregnant controls, *n* = 61 GDM, *n* = 26 non-pregnant mice on control diet, *n* = 13 non-pregnant mice on GDM diet. Source data are provided as a Source Data file. Data were analyzed by multiple two-tailed Mann-Whitney t-tests (a,c,e), or Wilcoxon matched-pairs signed rank test (b).

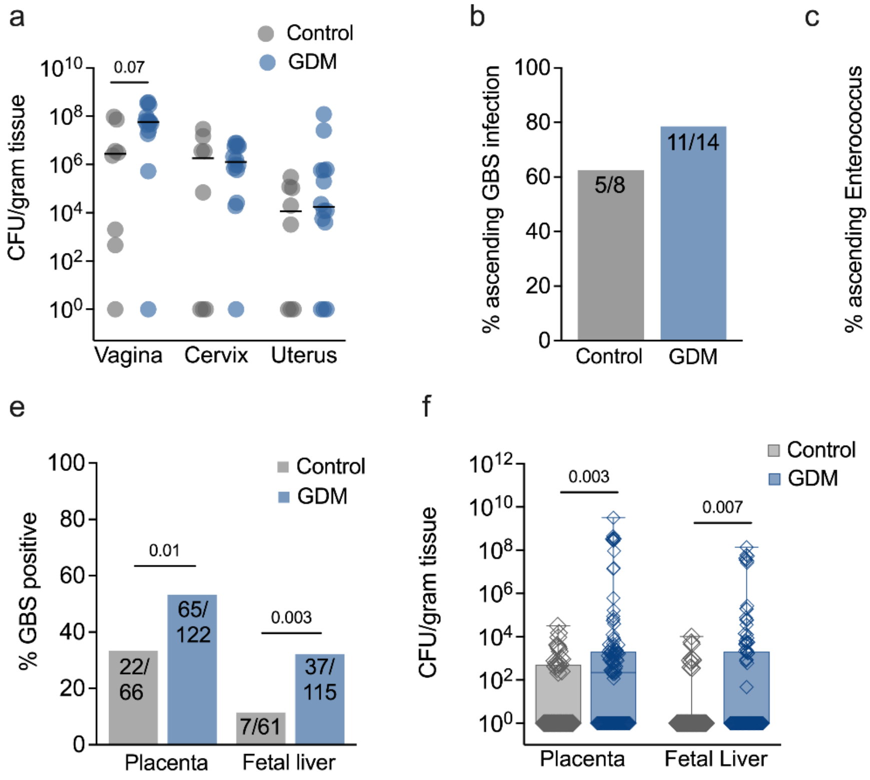

Supplementary Figure 2: Enhanced susceptibility of gestational diabetic mice to *in utero* group B Streptococcal fetal invasion is replicated by a GBS serotype V strain. GDM was induced via a high-fat high-sucrose (HFHS) diet followed by mid-gestational GBS vaginal colonization with GBS CNCTC 10/84 and tissue collection on E17.5. **a** GBS burden in maternal reproductive tract tissues. Proportion of dams with (**b**) ascending GBS infection, (**c**) ascending endogenous *Enterococcus*, or (**d**) fetal demise (e.g. reabsorption). **e** Percentage of placentae and fetal livers that were GBS positive, and (**f**) corresponding GBS burdens. **g** Percentage of placental-fetal units that had GBS detected in the placenta with no detection in the corresponding fetal liver. All data represent 3 independent replicates. Points represent individual samples and lines indicate medians (a,f). Box and whisker plots extend from 25th to 75th percentiles and show all points (f). Experimental numbers are n = 8 control and 14 GDM dams, and experimental numbers for placenta-fetal pairs in each group are given as denominators in E and G. Source data are provided as a Source Data file. Data was analyzed by two-tailed Mann-Whitney t-test (a,f) and two-sided Fisher's exact test (b-d, e, g).

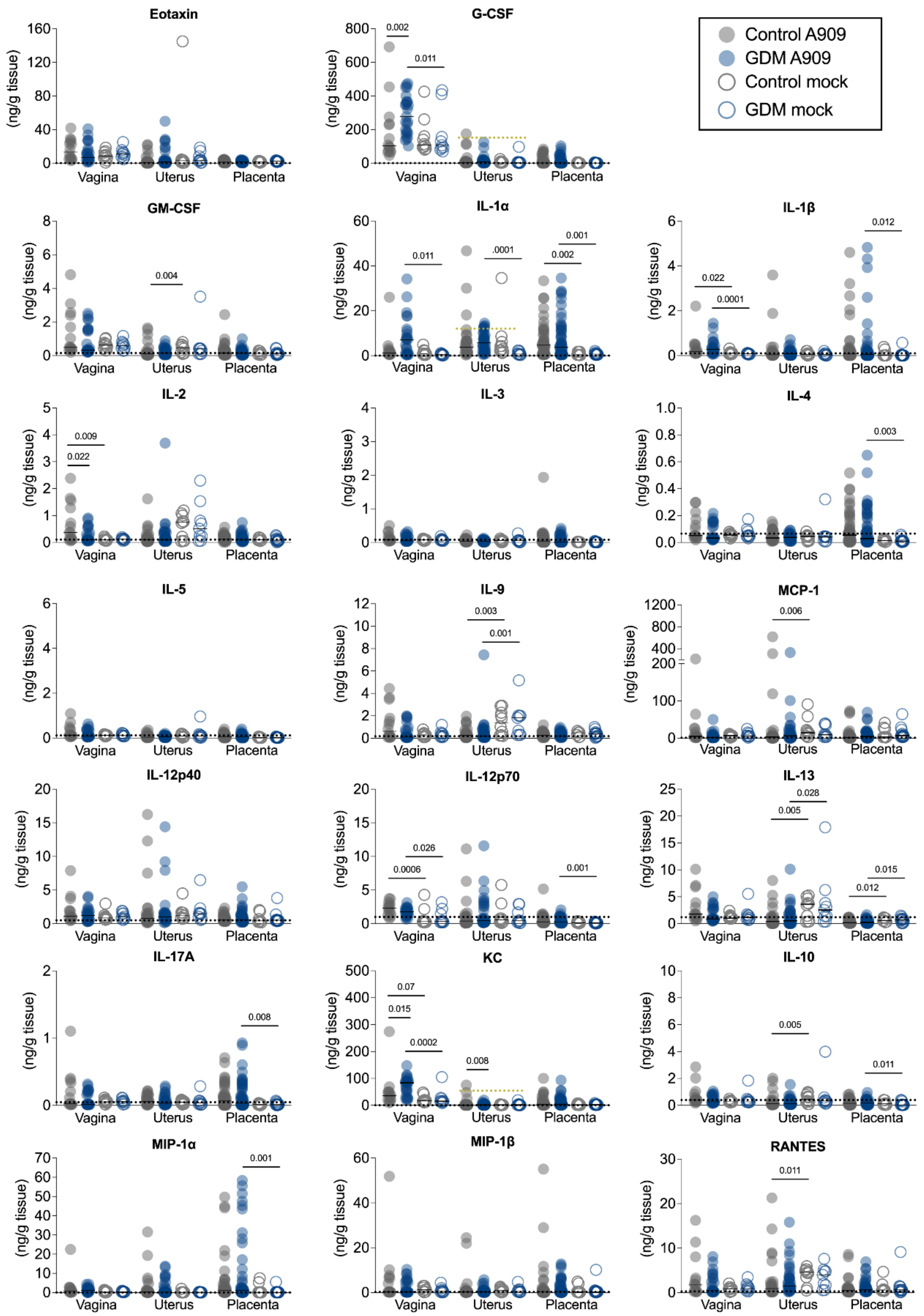

Supplementary Figure 3: Cytokine values from vaginal, uterine, and placental tissues. Quantification of 23 cytokines in tissues on E17.5 from pregnant controls and GDM mice that were inoculated with A909 or mock-infected, all of which are presented as a heatmap in Fig. 5a. Each point represents an independent mouse sample and data were analyzed by Kruskal-Wallis test followed by a two-stage linear step-up procedure of Benjamini, Krieger and Yekutieli to correct for multiple comparisons by controlling the false discovery rate (<0.05). Black dashed lines demarcate lower limit of detection, and yellow dashed lines indicate upper limit of detection (ULD) when sample(s) surpassed. Experimental *n* is provided in Fig. 5. Source data are provided as a Source Data file.

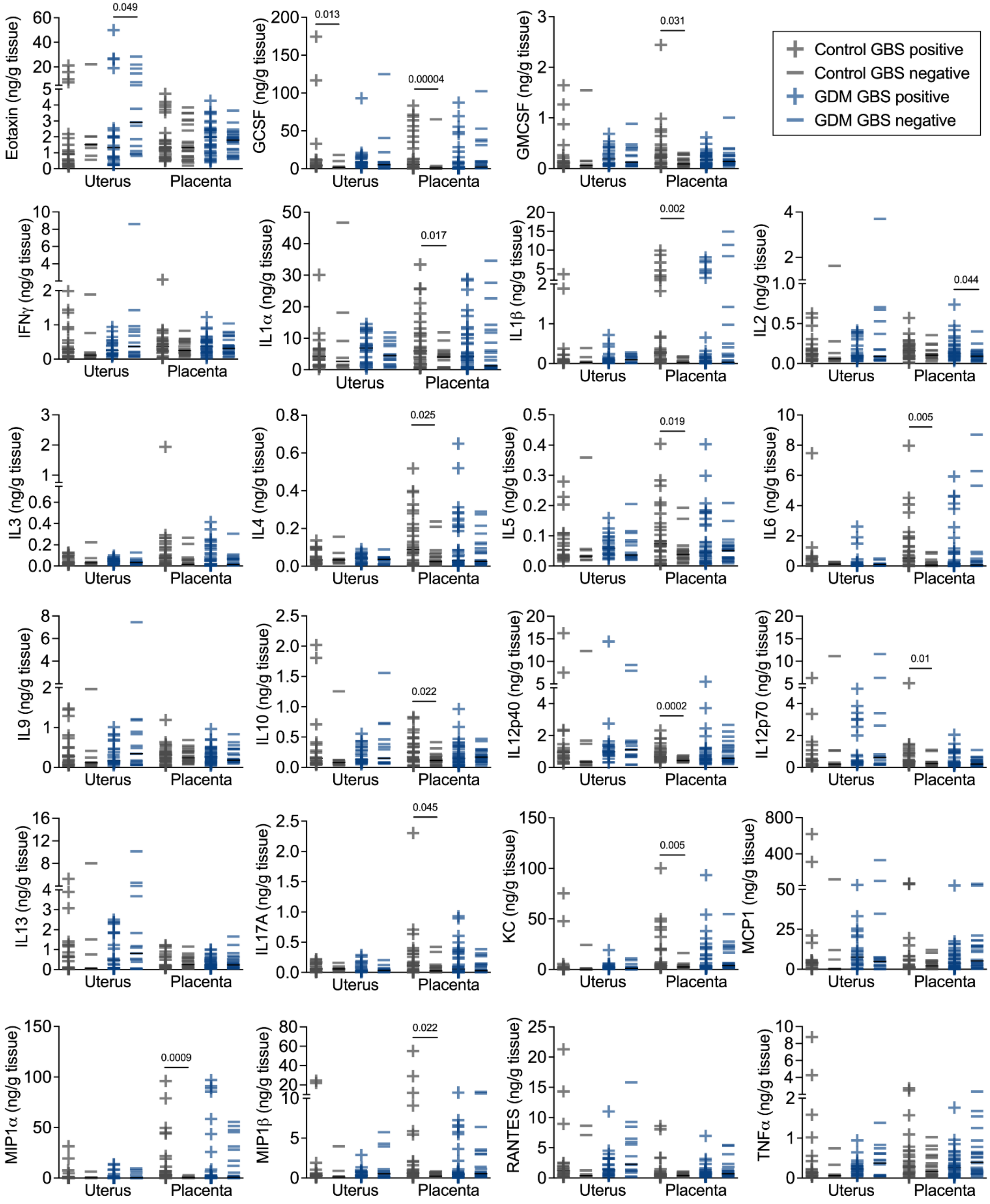

Supplementary Figure 4: Cytokine values from uterine, and placental tissues stratified by GBS presence or absence. Subgroup analysis of 23 cytokines in tissues on E17.5 from pregnant controls and GDM mice that were inoculated with A909 (shown in Fig 5a-b, Supplementary Fig. 3), with comparisons of GBS positive vs. negative tissues in each group. The sub-analysis is also presented as a heatmap in Fig. 5c. Each point represents an independent mouse sample and data were analyzed by two-tailed Mann-Whitney t-tests. Experimental *n* is provided in Fig. 5. Source data are provided as a Source Data file.

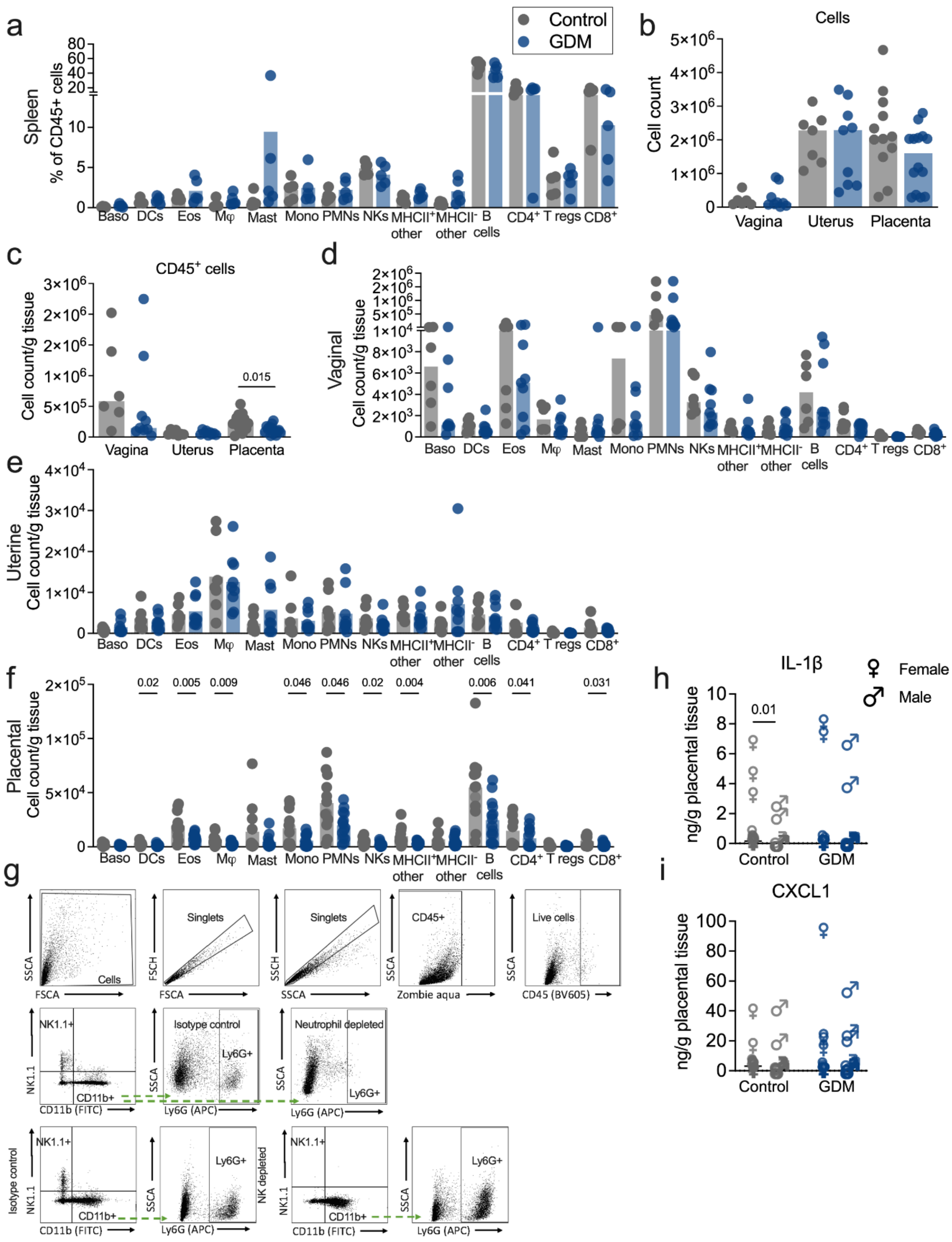

Supplementary Figure 5: Additional data for *in vivo* immune profiling experiments. a Immune cell frequencies in spleens from GBS-infected dams. **b** Total cell counts and (**c**) total live $CD45^+$ cell counts per tissue normalized to tissue weight. Immune cell counts in (**d**) vaginal, (**e**) uterine, and (**f**) placental tissues from GBS-infected dams. **g** Gating strategy for assessing depletion of NK cells or neutrophils with α-NK1.1 and α-Ly6G antibodies respectively. **h** IL-1β and (**i**) CXCL1 levels in placental tissues from E17.5 from GBS-infected dams, stratified by fetal sex. Data (a-f) are from 3 independent experiments with each point representing an individual mouse sample (*n* = 7 pregnant controls and 9 GDM, with 1-2 placentae per dam for a total of *n* = 12 control placentae and 14 GDM placentae). Data (h-i) are from 4 independent experiments, *n* = 15 female, *n* = 14 male associated control placentae, *n* = 19 female, *n* = 19 male associated GDM placentae. Source data are provided as a Source Data file. Data were analyzed by two-tailed Mann-Whitney t-tests. Related to Figures 5-7.

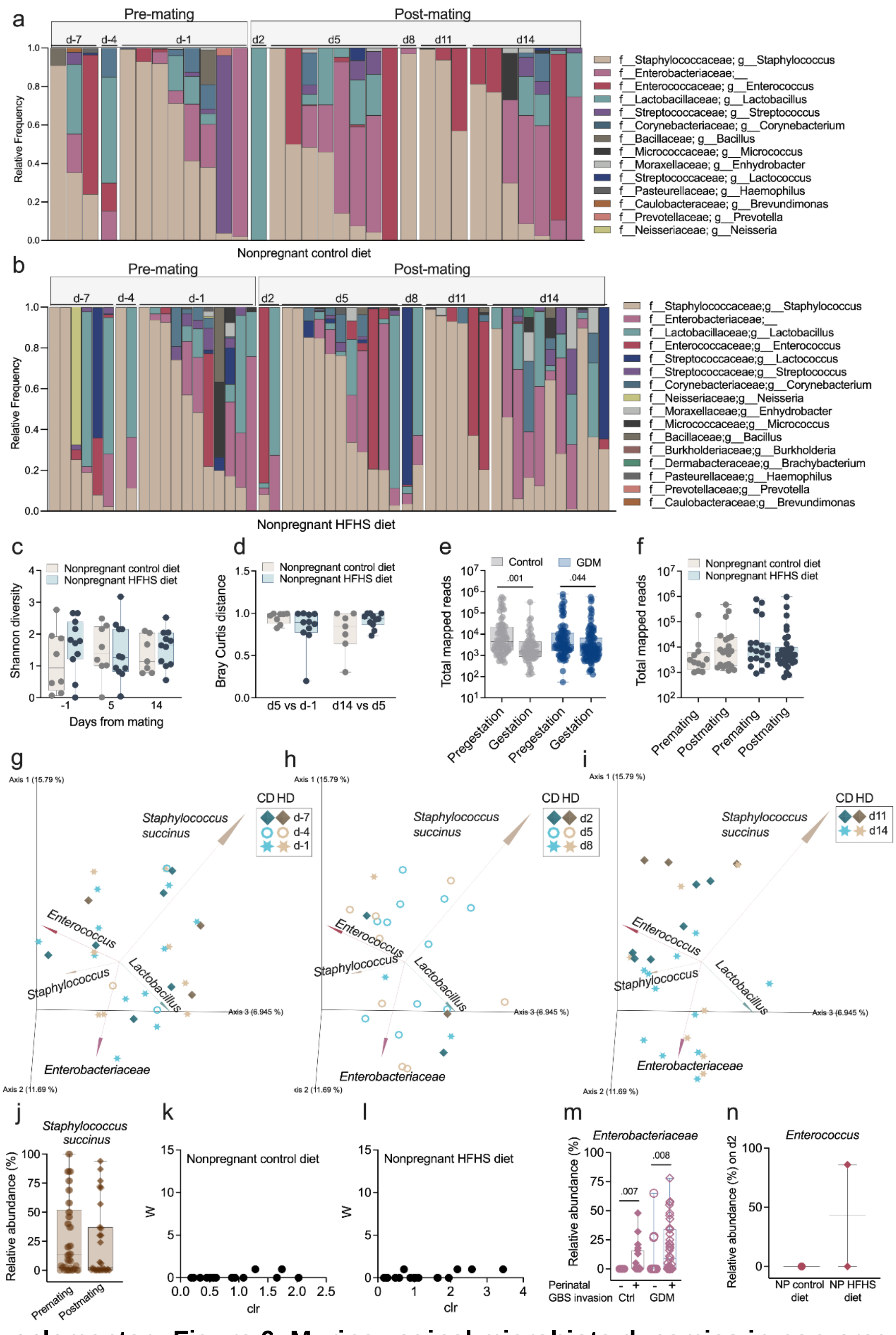

Supplementary Figure 6: Murine vaginal microbiota dynamics in non-pregnant cage mates on HFHS and control diets. a non-pregnant control-fed and (**b**) non-pregnant HFHS-fed mice were swabbed every three days from one week before mating (d-7) until d14 just prior to GBS challenge, and longitudinal vaginal microbiome composition was determined by 16Sv4 rRNA amplicon sequencing. **c** Shannon entropy of vaginal communities at d-1, d5, and d14 timepoints. **d** Bray Curtis distances between early (d-1 and d5) and late (d5 and d14) timepoints paired per mouse for non-pregnant mice, showing no effects of diet over the time course. **e** Total mapped reads for

pregestational vs. gestational timepoints in pregnant control and GDM groups. **f** Total mapped reads for premating and postmating timepoints in nonpregnant mice on control or HFHS diets. Bray Curtis distance matrix principle coordinate analysis (PcoA) plots of vaginal communities in non-pregnant mice on control diet (CD) or high-fat high-sucrose diet (HD) at (**g**) pre-mating, (**h**) early, and (**i**) mid-experimental timepoints. **j** Relative abundance of *S. succinus* at pre-mating and post-mating timepoints with control and HFHS samples combined. Analysis of Composition of Microbes (ANCOM) in non-pregnant mice on (**k**) control or (**l**) HFHS diet across all timepoints. **m** Relative abundances of *Enterobacteriaceae* at early gestation (E2, E5, E8) timepoints in pregnant control and GDM dams, stratified by GBS dissemination showing earlier effects in line with later timepoint data shown in Fig. 9. **n** *Enterococcus* relative abundance 2 days after mating, showing GDM specific effects compared to data in Fig. 9. Points represent individual samples and lines indicate medians. Box and whisker plots extend from 25th to 75th percentiles and show all points (c,d,k-n). Data are from 4 independent experiments, n = 8 non-pregnant mice on control diet, n = 11 non-pregnant mice on HFHS diet. Experimental numbers for pregnant mice are given in Fig. 9. Source data are provided as a Source Data file. Data were analyzed by two-tailed Mann-Whitney t-test (c-f, j, m-n).

Supplementary Table 1: Differential gene expression of GBS in murine uterine vs. vaginal tissue. Related to Figure 2. Data were analyzed by Wald tests in the DESeq2 pipeline.

Current gene locus	Former locus tag	Gene name	Expected product	Pregnant control log_2 Fold-Change (*p*-value)[a]		GDM log_2 Fold-Change (*p*-value)[b]	
				Exp 1	*Exp 1 & 2*	*Exp 1*	*Exp 1 & 2*
SAK_RS07995	SAK_1585		acetyltransferase	**3.61** (9.07E-34)	**4.47** (8.25E-78)	**3.95** (1.62E-43)	**4.49** (1.76E-78)
SAK_RS10655	SAK_2115		GRP family sugar transporter	**3.57** (0.025)	**2.79** (0.04)	1.24 (1)	1.54 (1)
SAK_RS00885	SAK_0179		L-lactate dehydrogenase	**2.83** (4.76E-12)	**4.43** (4.6E-28)	**3.17** (2.90E-17)	**4.42** (2.00E-25)
SAK_RS10730	SAK_2130	*yfhO*	YfhO family protein	**2.39** (0.048)	1.47 (1)	0.77 (1)	0.52 (1)
SAK_RS09065	SAK_1801	*rsmA*	ribosomal RNA small subunit methyltransferase A	**2.37** (7.45E-07)	1.31 (1)	**2.55** (5.35E-09)	1.36 (1)
SAK_RS00825	SAK_0167	*rbsC*	ribose ABC transporter permease	**2.16** (1.77E-17)	**2.59** (1.58E-22)	**2.28** (1.15E-21)	**2.98** (3.06E-35)
SAK_RS02820	SAK_0566	*bioB*	biotin synthase	**1.87** (1.20E-05)	1.04 (1)	**1.88** (9.87E-06)	1.04 (1)
SAK_RS08150	SAK_1616		YneF family protein	1.09 (1)	**1.80** (1.16E-04)	1.03 (0.7)	**1.61** (.022)
SAK_RS02870	SAK_0576		DUF3165 family protein	**1.75** (0.044)	0.74 (1)	1.65 (0.22)	0.79 (1)
SAK_RS04330	SAK_0866		DUF3270 domain-containing protein	1.29 (.14)	**1.71** (8.6E-09)	**1.33** (0.049)	**1.61** (1.49E-06)
SAK_RS00940	SAK_0189	*bgrR*	response regulator transcription factor BgrR	1.05 (1)	**1.54** (7.29E-05)	1.27 (0.51)	**1.52** (1.40E-04)
SAK_RS08980	SAK_1784		CHAP domain-containing protein	**1.38** (0.00005)	**1.81** (2.29E-07)	**1.28** (0.018)	**1.82** (9.44E-08)
SAK_RS08695	SAK_1729		hypothetical protein	**-1.31** (0.0004)	-0.82 (1)	**-1.23** (0.042)	-0.87 (1)
SAK_RS01760	SAK_0355	*proB*	glutamate 5-kinase	**-1.34** (0.0002)	-0.59 (1)	**-1.32** (0.0010)	-0.52 (1)

SAK_RS10120	SAK_2012	*mrvR*	GntR family transcriptional regulator	**-1.34** (2.86 E-07)	-0.76 (1)	-1.11 (1)	-0.62 (1)
SAK_RS08640	SAK_1718		DUF1129 family protein	**-1.37** (0.02 8)	-1.19 (.36)	-1.22 (1)	-1.15 (1)
SAK_RS08460	SAK_1680	*gatA*	glutamyl-tRNA(Gln) amidotransferase subunit A	**-1.44** (2.86 E-07)	-0.62 (1)	-1.13 (1)	-0.35 (1)
SAK_RS09300	SAK_1847		N-acetyltransferase	**-1.45** (1.20 E-06)	-0.8 (1)	-1.19 (1)	-0.66 (1)
SAK_RS10715	SAK_2127		YoaK family protein	-1.29 (1)	-1.05 (1)	**-1.50** (0.005)	-1.25 (0.46)
SAK_RS02315	SAK_0465	*perR*	peroxide-responsive transcriptional repressor	**-1.47** (0.00 5)	-1.36 (0.25)	**-1.56** (0.000 22)	**-1.41** (0.049)
SAK_RS03800	SAK_0763		Recombinase family protein	-0.54 (1)	-1.19 (1)	-1.03 (1)	**-1.63** (0.011)
SAK_RS07530	SAK_1496		CPBP family intramembrane glutamic endopeptidase	-1.13 (1)	**-1.57** (0.005)	-0.96 (1)	**-1.70** (5.99E -05)
SAK_RS09900	SAK_1967		aminoglycoside 6-adenylyltransfera se	-0.97 (1)	**-1.62** (.003)	-1.22 (1)	**-1.71** (1.45E -04)
SAK_RS01595	SAK_0323		hypothetical protein	**-1.71** (0.01 8)	-0.89 (1)	**-2.05** (4.50E -06)	-1.06 (1)
SAK_RS07010	SAK_1393		ABC transporter ATP-binding protein	**-1.80** (3.74 E-18)	-0.92 (1)	**-1.62** (2.09E -10)	-0.75 (1)
SAK_RS03260	SAK_0651		hypothetical protein	-1.59 (0.76)	**-1.98** (1.04E -06)	-1.69 (0.19)	**-2.09** (1.57E -08)
SAK_RS06220	SAK_1240		DUF2969 domain-containing protein	**-2.51** (1.30 E-31)	-0.62 (1)	**-2.46** (9.07E -30)	-1.35 (1)
SAK_RS06520	SAK_1300		DUF3042 family protein	**-2.96** (4.75 E-87)	**-2.82** (3.21E -60)	**-2.77** (4.36E -71)	**-2.83** (1.08E -60)
SAK_RS11770	NA[c]		hypothetical protein	ND[d]	**-5.81** (7.97E -46)	ND	**-6.17** (2.27E -53)

[a]Fold-change between GBS from uterine tissue vs. GBS from vaginal tissue in pregnant control mice
[b]Fold-change between GBS from uterine tissue vs. GBS from vaginal tissue in GDM mice
[c]NA = Not applicable
[d]ND = Not detected

Supplementary Table 2: Differential gene expression of GBS in murine placental vs. uterine tissue. Related to Figure 2. Data were analyzed by Wald tests in the DESeq2 pipeline.

**Current gene locus**	**Former locus tag**	**Gene name**	**Expected product**	**Pregnant control $\log_2$ Fold-Change (*p*-value)[a]**	**GDM $\log_2$ Fold-Change (*p*-value)[b]**
SAK_RS08960	SAK_1780	*rimI*	ribosomal protein S18-alanine N-acetyltransferase	4.91 (1.65E-81)	4.91 (1.22E-81)
SAK_RS05065	SAK_1012		TIGR02328 family protein	4.05 (1.06E-80)	4.07 (1.22E-81)
SAK_RS09775	SAK_1941		hypothetical protein	3.54 (7.78E-81)	3.28 (7.71E-65)
SAK_RS03100	SAK_0620		bifunctional DNA primase/polymerase	3.35 (5.64E-58)	3.03 (3.39E-43)
SAK_RS08155	SAK_1617		hypothetical protein	3.17 (1.62E-47)	3.17 (3.48E-47)
SAK_RS04225	SAK_0844		amino acid ABC transporter ATP-binding protein	3.11 (2.23E-41)	2.62 (3.46E-24)
SAK_RS08905	SAK_1768		GNAT family N-acetyltransferase	3.00 (5.93E-50)	3.00 (2.09E-49)
SAK_RS02610	SAK_0525		PTS sugar transporter subunit IIB	2.94 (4.8E-97)	2.95 (4.1E-98)
SAK_RS09935	SAK_1974		ABC transporter permease	2.37 (1.6E-38)	2.19 (3.78E-29)
SAK_RS06105	SAK_1218		DNA alkylation repair protein	2.27 (1.15E-17)	2.05 (5.94E-12)
SAK_RS06540	SAK_1304		uracil-DNA glycosylase family protein	2.26 (5.03E-26)	1.98 (1.93E-15)
SAK_RS05750	SAK_1150		NADH-dependent flavin oxidoreductase	2.20 (1.36E-23)	1.95 (1.34E-14)
SAK_RS08605	SAK_1709		hypothetical protein	2.19 (4.41E-21)	1.93 (9.17E-13)
SAK_RS06190	SAK_1234		LrgB family protein	0.86 (1)	2.14 (5.01E-03)
SAK_RS05715	SAK_1143	*cls*	cardiolipin synthase	1.94 (3.49E-23)	1.67 (8.16E-12)
SAK_RS10565	SAK_2097	*dnaB*	replicative DNA helicase	1.93 (3.11E-13)	1.82 (2.87E-10)
SAK_RS04015	SAK_0805		hypothetical protein	1.88 (3.21E-10)	1.59 (1.62E-04)
SAK_RS06335	SAK_1263		LysR family transcriptional regulator	1.85 (2.11E-03)	1.18 (1)
SAK_RS02825	SAK_0567		hypothetical protein	1.82 (1.15E-03)	0.99 (1)
SAK_RS11275	SAK_2004	*rpmG*	50S ribosomal protein L33	1.77 (1.78E-06)	1.69 (4.69E-05)
SAK_RS09625	SAK_1911		PTS mannose transporter subunit IIA	1.65 (2.34E-04)	1.53 (9.71E-03)

SAK_RS11740	SAK_2017		ABC transporter substrate-binding protein	1.63 (7.10E-06)	1.60 (3.07E-05)
SAK_RS10445	SAK_2076		hypothetical protein	1.59 (3.64E-19)	1.56 (1.47E-17)
SAK_RS02015	SAK_0406		DEAD/DEAH box helicase	1.54 (8.25E-09)	1.43 (1.12E-05)
SAK_RS05190	SAK_1037	*pyk*	pyruvate kinase	1.49 (4.92E-08)	1.62 (6.24E-13)
SAK_RS01175	SAK_0239	*proC*	pyrroline-5-carboxylate reductase	1.46 (2.70E-08)	1.32 (4.24E-04)
SAK_RS03140	SAK_0628		hypothetical protein	1.43 (7.60E-03)	1.23 (1)
SAK_RS08995	SAK_1787		aromatic acid exporter family protein	1.39 (2.95E-03)	1.22 (0.86)
SAK_RS05570	SAK_1113		hypothetical protein	1.37 (1.15E-03)	1.26 (0.15)
SAK_RS05325	SAK_1065		GNAT family N-acetyltransferase	1.36 (3.81E-02)	1.54 (0.000044)
SAK_RS01005	SAK_0203	*sufB*	Fe-S cluster assembly protein SufB	1.32 (5.59E-05)	1.27 (1.60E-03)
SAK_RS10215	SAK_2030		IreB family regulatory phosphoprotein	1.31 (0.03)	1.51 (2.16E-06)
SAK_RS07735	SAK_1535	*cvfB*	RNA-binding virulence regulatory protein CvfB	1.22 (2.34E-04)	1.47 (5.71E-20)
SAK_RS04415	SAK_0883		DUF4430 domain-containing protein	-1.37 (0.03)	-1.59 (4.30E-06)
SAK_RS05930	SAK_1185		redox-sensing transcriptional repressor Rex	-1.41 (3.57E-11)	-1.49 (7.96E-16)
SAK_RS08170	SAK_1620		HD domain-containing protein	-1.47 (1.88E-04)	-1.49 (7.69E-05)
SAK_RS08295	SAK_1646		energy-coupling factor transporter transmembrane component T	-1.49 (1.32E-07)	-1.66 (4.66E-14)
SAK_RS09245	SAK_1836		membrane protein	-1.53 (2.75E-05)	-1.68 (8.28E-09)
SAK_RS07620	SAK_1513	*secG*	preprotein translocase subunit SecG	-1.42 (0.88)	-1.63 (0.03)
SAK_RS08750	SAK_1740		MIP/aquaporin family protein	-1.64 (3.75E-04)	-1.47 (0.038)
SAK_RS01900	SAK_0383	*gmk*	guanylate kinase	-1.61 (0.06)	-1.66 (0.02)
SAK_RS04815	SAK_0962	*tenA*	thiaminase II	-1.76 (5.93E-06)	-1.69 (9.75E-05)
SAK_RS09540	SAK_1895		PTS sugar transporter subunit IIA	-1.80 (4.17E-07)	-1.57 (1.81E-03)
SAK_RS00940	SAK_0189	*bgrR*	response regulator transcription factor BgrR	-1.84 (3.05E-12)	-1.81 (3.14E-11)

SAK_RS00825	SAK_0167	*rbsC*	ribose ABC transporter permease	-1.87 (5.93E-06)	-2.01 (4.41E-08)
SAK_RS06790	SAK_1349		DUF1836 domain-containing protein	-1.90 (5.06E-14)	-1.70 (2.33E-08)
SAK_RS08150	SAK_1616		YneF family protein	-2.13 (2.42E-09)	-1.79 (1.96E-04)
SAK_RS07765	SAK_1541		ABC transporter permease	-2.87 (3.94E-52)	-2.88 (4.53E-53)
SAK_RS07995	SAK_1585		acetyltransferase	-4.90 (9E-88)	-5.01 (9.14E-93)
SAK_RS00885	SAK_0179		L-lactate dehydrogenase	-5.05 (3.77E-32)	-5.21 (1.06E-34)

[a]Fold-change between GBS from placental tissue vs. GBS from uterine tissue in pregnant control mice
[b]Fold-change between GBS from placental tissue vs. GBS from uterine tissue in GDM mice

Supplementary Table 3: DEGs of GDM vs. pregnant control reproductive tissues. Related to Figure 3. Data were analyzed by Wald tests in the DESeq2 pipeline.

Gene	Expected function(s)	$\log_2$ Fold-Change (*p*-value)
**GDM vaginal vs. pregnant control vaginal tissue**		*Exp 1 and 2*
Tmem276-zftraf1	Predicted uncharacterized protein.	31.03 (3.09E-39)
Gm24924	Small nuclear RNA.	24.49 (8.28E-11)
Chil4	Chitin binding activity and chitin catabolic processes; Kinase binding activity; Positive regulator of chemokine production.	-6.14 (1.33E-05)
Eif2s3y	Eukaryotic translation initiation factor 2, subunit 3, structural gene Y-linked. Predicted to facilitate formation of the translation preinitiation complex.	-14.73 (9.79E-09)
Ctrb1	Chymotrypsinogen B1.Predicted to promote proteolysis in lysosomes.	-14.85 (1.09E-08)
Mug2	Murinoglobulin 2. Predicted to negatively regulate peptidase activity.	-15.76 (2.67E-20)
Lhcgr	Luteinizing hormone/choriogonadotropin receptor. Mediates many biological processes including reproductive organ development, and steroid hormone biosynthesis.	-16.74 (1.00E-10)
Mug-ps1	Murinoglobulin, pseudogene 1.	-18.07 (6.44E-05)
Sbpl	Predicted to have activity in extracellular space. Orthologous to human zymogen granule protein 16B which is predicted to mediate carbohydrate binding.	-32.80 (1.41E-06)
**GDM uterine vs. pregnant control uterine tissue**		
Col17a1	Collagen, type XVII, alpha 1. Important in organization of extracellular matrix.	-3.04 1.70E-02
Pitx1	Paired-like homeodomain transcription factor 1. Positively regulates transcription by RNA polymerase II.	-3.30 4.23E-02
Grhl3	Grainyhead like transcription factor 3. Promotes activity of DNA-binding transcription factors.	-3.70 4.68E-02
Krt5	Keratin 5. Predicted to enable binding activity of scaffold proteins.	-4.01 2.06E-02
Gm20554	Noncoding RNA.	-4.73 1.24E-02
Ces2a	Carboxylesterase 2A. Predicted to be located in the endoplasmic reticulum and to promote carboxylic ester hydrolase activity. Acts upstream of protein glycosylation.	-4.77 1.70E-02

Col2a1	Collagen, type II, alpha 1. Encodes the alpha-1 subunit of type II collagen.	-4.90<br>4.91E-02
Mug2	Murinoglobulin 2. Predicted to negatively regulate peptidase activity.	-6.29<br>2.31E-02
Bhmt	Betaine-homocysteine methyltransferase. Involved in methionine biosynthetic processes.	-6.91<br>2.16E-02
Lhcgr	Luteinizing hormone/choriogonadotropin receptor. Mediates many biological processes including reproductive organ development, and steroid hormone biosynthesis.	-8.94<br>4.61E-02
Rln1	Relaxin 1. Regulates nitric oxide signal transduction, apoptosis, spermatogenesis and mammary gland development.	-10.38<br>4.23E-02
Serpina6	Serine (or cysteine) peptidase inhibitor, clade A, member 6. Major blood transport protein for glucocorticoids and progestins.	-11.64<br>3.01E-07
Krt77	Keratin 77. Structural protein located in cytoskeleton.	-25.10<br>1.66E-32
Cst8	Predicted to negatively regulate peptidase activity Expression specific to the reproductive tract, suggesting a role in reproduction.	-25.23<br>1.24E-02
**GDM placental vs. pregnant control placental tissue**		
Akr1cl	Aldo-keto reductase family 1, member C-like. Predicted to be involved in glycoside metabolism, progesterone and prostaglandin metabolic processes.	-15.370<br>(6.72E-13)
Padi6	Peptidyl arginine deiminase, type VI. Involved in cytoplasm and cytoskeletal organization. Human ortholog implicated in infertility.	-15.371<br>(3.16E-10)
Try4	Trypsin 4. Predicted to facilitate proteolysis and calcium ion binding activities. Located in extracellular matrix.	27.383<br>(1.90E-03)
