# Supplementary material for: Gestational diabetes augments group B Streptococcus infection by disrupting maternal immunity and the vaginal microbiota
Source: Nat Commun. 2024 Feb 3;15:1035. doi: 10.1038/s41467-024-45336-6 (PMC10838280; doi:10.1038/s41467-024-45336-6)
Supplement: Supplementary file 3 — Reporting Summary [file 41467_2024_45336_MOESM3_ESM.pdf]

nature portfolio

Corresponding author(s): Kathryn Patras

Last updated by author(s): Jan 15, 2024

Reporting Summary

Nature Portfolio wishes to improve the reproducibility of the work that we publish. This form provides structure for consistency and transparency in reporting. For further information on Nature Portfolio policies, see our Editorial Policies and the Editorial Policy Checklist.

Statistics

For all statistical analyses, confirm that the following items are present in the figure legend, table legend, main text, or Methods section.

n/a	Confirmed	
☐	☒	The exact sample size (*n*) for each experimental group/condition, given as a discrete number and unit of measurement
☐	☒	A statement on whether measurements were taken from distinct samples or whether the same sample was measured repeatedly
☐	☒	The statistical test(s) used AND whether they are one- or two-sided<br>*Only common tests should be described solely by name; describe more complex techniques in the Methods section.*
☐	☒	A description of all covariates tested
☐	☒	A description of any assumptions or corrections, such as tests of normality and adjustment for multiple comparisons
☐	☒	A full description of the statistical parameters including central tendency (e.g. means) or other basic estimates (e.g. regression coefficient) AND variation (e.g. standard deviation) or associated estimates of uncertainty (e.g. confidence intervals)
☐	☒	For null hypothesis testing, the test statistic (e.g. *F*, *t*, *r*) with confidence intervals, effect sizes, degrees of freedom and *P* value noted<br>*Give P values as exact values whenever suitable.*
☒	☐	For Bayesian analysis, information on the choice of priors and Markov chain Monte Carlo settings
☐	☒	For hierarchical and complex designs, identification of the appropriate level for tests and full reporting of outcomes
☒	☐	Estimates of effect sizes (e.g. Cohen's *d*, Pearson's *r*), indicating how they were calculated

Our web collection on statistics for biologists contains articles on many of the points above.

Software and code

Policy information about availability of computer code

Data collection	The code is accessible at GitHub under project "Transcriptional-and-vaginal-microbial-analyses-in-a-mouse-model-of-gestational-diabetes-" and deposited in Zenodo [https://zenodo.org/records/10505424] under doi.org/10.5281/zenodo.10505424.
Data analysis	For bacterial RNA sequences, quality control and adapter trimming was performed with bcl2fastq, read mapping was performed with HISAT2, and read quantification was performed using Subread's featureCounts, with alignment to the A909 reference genome (NCBI Nucleotide accession number NC_007432.1). For murine RNA sequences, quality control and adapter trimming were performed with bcl-convert. Read mapping was performed via STAR to the mm10 version of the mouse genome and feature quantification was performed using RSEM. Raw counts normalization and differential expression analyses were performed using R package DESeq2 (v 1.40.1)138. RStudio (2022.12.0+353) was used for visualizations. The fGSEA R package (v1.20.0, RRID:SCR_020938) was used for gene set enrichment analyses with 10,000 permutations, a minimum gene set of 15, a maximum gene set of 500, and the Reactome gene set collections from the Molecular Signatures Database. For structural predictions, software used included AlphaFold2, visualization in ChimeraX 1.6.1 and HHpred server. For microbiome analyses, raw data files were converted into FASTQs and demultiplexed using the Illumina 'bcl2fastq' software and single-index barcodes. Demultiplexed read pairs underwent initial quality filtering using bbduk.sh (BBMap version 38.82, 5) removing Illumina adapters, PhiX reads and reads with a Phred quality score below 15 and length below 100 bp after trimming. Quality controlled reads were merged using bbmerge.sh (BBMap version 38.82), and further filtered using vsearch with a maximum expected error of 0.05, maximum length of 254 bp and minimum length of 252 bp. Sequences were joined and trimmed using Deblur through the DADA2 plugin on QIIME2 v2021.11 with taxonomic assignments using naïve bayes sklearn classifier trained on the GreenGenes OTUs database 13_8 and further processed through the Decontam R package.

For manuscripts utilizing custom algorithms or software that are central to the research but not yet described in published literature, software must be made available to editors and reviewers. We strongly encourage code deposition in a community repository (e.g. GitHub). See the Nature Portfolio guidelines for submitting code & software for further information.

Data

Policy information about availability of data

All manuscripts must include a data availability statement. This statement should provide the following information, where applicable:

- Accession codes, unique identifiers, or web links for publicly available datasets
- A description of any restrictions on data availability
- For clinical datasets or third party data, please ensure that the statement adheres to our policy

The 16Sv4 rRNA sequencing data generated in this study have been deposited in NCBI Sequence Read Archive under BioProject accession number PRJNA988548 [https://www.ncbi.nlm.nih.gov/bioproject/?term=PRJNA988548]. The A909 reference genome used in this study is publicly available on NCBI Nucleotide under accession number NC_007432.1 [https://www.ncbi.nlm.nih.gov/nuccore/NC_007432.1]. The RNA sequencing data generated in this study have been deposited in NCBI Gene Expression Omnibus under GEO accession number GSE236335 [https://www.ncbi.nlm.nih.gov/geo/query/acc.cgi?acc=GSE236335] and BioProject accession number PRJNA990648 [https://www.ncbi.nlm.nih.gov/bioproject/?term=PRJNA990648]. Other source data used to generate figures are provided with this paper as a Source Data file.

Research involving human participants, their data, or biological material

Policy information about studies with human participants or human data. See also policy information about sex, gender (identity/presentation), and sexual orientation and race, ethnicity and racism.

Reporting on sex and gender	N/A
Reporting on race, ethnicity, or other socially relevant groupings	N/A
Population characteristics	N/A
Recruitment	N/A
Ethics oversight	N/A

Note that full information on the approval of the study protocol must also be provided in the manuscript.

Field-specific reporting

Please select the one below that is the best fit for your research. If you are not sure, read the appropriate sections before making your selection.

☒ Life sciences ☐ Behavioural & social sciences ☐ Ecological, evolutionary & environmental sciences

For a reference copy of the document with all sections, see nature.com/documents/nr-reporting-summary-flat.pdf

Life sciences study design

All studies must disclose on these points even when the disclosure is negative.

Sample size	Power calculations were performed using G*Power and variance extrapolation from previous results, with a power of 0.8, P-value = 0.05, and effect size of 0.4-0.8.
Data exclusions	No data was excluded from analyses.
Replication	All experiments were performed in at least two independent replicate experiments with data combined prior to statistical analyses. Numbers of experimental replicates are provided in figure legends.
Randomization	Animals were randomized into cages by vivarium staff and randomly assigned to treatment groups prior to experiment initiation.
Blinding	Blinding was not performed as a part of this study since group identifiers, such as diet, were visible during animal handling and sample collection.

Reporting for specific materials, systems and methods

We require information from authors about some types of materials, experimental systems and methods used in many studies. Here, indicate whether each material, system or method listed is relevant to your study. If you are not sure if a list item applies to your research, read the appropriate section before selecting a response.

Materials & experimental systems

n/a	Involved in the study
☐	☒ Antibodies
☒	☐ Eukaryotic cell lines
☒	☐ Palaeontology and archaeology
☐	☒ Animals and other organisms
☒	☐ Clinical data
☒	☐ Dual use research of concern
☒	☐ Plants

Methods

n/a	Involved in the study
☒	☐ ChIP-seq
☐	☒ Flow cytometry
☒	☐ MRI-based neuroimaging

Antibodies

Antibodies used

For flow cytometry experiments, each antibody was added at 0.25 µL/sample for all antibodies except for anti-CD23, anti-CD24, and anti-Ly-6C for which 0.125 µL was added, and anti-CD3 for which 0.5 µL was added. Antibodies were combined with 10 µL brilliant stain buffer plus (cat. 566385, BD Biosciences) per sample and brought up to a total volume of 50 µL/sample with cell staining buffer (cat. 420201, BioLegend). Antibodies used include Fc block (CD16/CD32, clone 2.4G2, BD Biosciences, cat. 553141), anti-CD3 (BV480, clone 145-2C11, cat. 746368, BD Biosciences), anti-CD45 (BV605, clone 30-F11, cat. 563053, BD Biosciences), anti-CD11b (APC-Cy7, clone M1/70, cat. 561039), anti-CD11c (BV785, clone N418, cat. 117335, BioLegend), anti-Ly6G (AF700, clone 1A8, cat. 561236, BD Biosciences), anti-NK1.1 (BUV737, clone PK136, cat. 741715, BD Biosciences), anti-CD19 (BB515, clone 1D3, cat. 564509, BD Biosciences), anti-CD8a (PE-Cy7, clone QA17A07, cat. 155018, BioLegend), anti-CD4 (BUV563, clone GK1.5, cat. 612923, BD Biosciences), anti-CD23 (BUV395, clone B3B4, cat. 740216, BD Biosciences), anti-CD44 (BUV805, clone IM7, cat. 741921, BD Biosciences), anti-CD64 (BV421, clone X54-5/7.1, cat. 139309, BioLegend), anti-MHCII (BV650, clone M5/114.15.2, cat. 563415, BD Biosciences), anti-CD24 (BV711, clone M1/69, cat. 563450, BD Biosciences), anti-CD62L (PE, clone M1/69, cat. 161204, BioLegend), anti-CD69 (PE-CF594, clone H1.2F3, cat. 562455, BD Biosciences), anti-Ly-6C (PerCP-Cy5.5, clone HK1.4, cat. 128012, BioLegend), anti-CD25 (APC, clone PC61, cat. 557192, BD Biosciences).
For immune depletion experiments, animals received two i.p. injections 48 h apart of 250 µg (in 100 µL) of one of the following: anti-NK1.1 antibody (Bioxcell, clone PK136, cat. BE0036, Lot 828622A2), mouse IgG2a isotype control (Bioxcell, clone C1.18.4, cat. BE0085, Lot 833922A2), anti-Ly6G antibody (Bioxcell, clone 1A8, cat. BE0075-1, Lot 807722M1), and rat IgG2a isotype control (Bioxcell, clone 2A3, cat. BE0089, Lot 849322J2).

Validation

All antibodies are used in this manuscript are well-established, commercially available antibodies. Validations for each of these commercial antibodies are provided on the manufacturers' websites and include specificity and quality control testing via flow cytometry and testing for contaminants including endotoxin. Bioxcell antibodies were purified from tissue culture supernatant in an animal free facility and endotoxin levels determined by LAL gel clotting assay.

Animals and other research organisms

Policy information about studies involving animals; ARRIVE guidelines recommended for reporting animal research, and Sex and Gender in Research

Laboratory animals

Wild-type (WT) female and male C57BL/6J mice aged 6 weeks were purchased from Jackson Laboratories (strain code 000664). Control and GDM groups were assigned randomly and mice were housed at 3 animals per cage. Mice ate and drank ad libitum and had a 12h light cycle per day with housing conditions at maintained at 68–72°F and 30–70% humidity.

Wild animals

Study did not involve wild animals.

Reporting on sex

Male mice were used solely for mating and female mice were used for all in vivo experiments. Fetal and pup sexes were determine by PCR and data disaggregated as indicated in the figures.

Field-collected samples

Study did not involve field-collected samples.

Ethics oversight

All animal protocols and procedures were approved by the BCM Institutional Animal Care and Use Committee.

Note that full information on the approval of the study protocol must also be provided in the manuscript.

Flow Cytometry

Plots

Confirm that:

☒ The axis labels state the marker and fluorochrome used (e.g. CD4-FITC).

☐ The axis scales are clearly visible. Include numbers along axes only for bottom left plot of group (a 'group' is an analysis of identical markers).

☐ All plots are contour plots with outliers or pseudocolor plots.

☒ A numerical value for number of cells or percentage (with statistics) is provided.

Methodology

Sample preparation	Vaginal and placental tissues were transected with one-third processed for burden quantification and the remaining two-thirds processed for flow cytometric quantification of immune cell populations. For uterine tissues, each horn was transected in half, and the halves from each horn were pooled and processed for either burden quantification as described above, or for immune cell quantification. Tissues harvested for flow were placed in 450 µL (placenta) or 900 µL (vagina, uterus) of RPMI, and mechanically disrupted with scissors until ~90% of sample was fine enough to pass through a p1000 tip. Collagenase (0.2mg/mL) and DNase (50 U/mL) were added to each vaginal and uterine sample, and Collagenase (0.2mg/mL) and DNase (50 U/mL) were added to placental samples. After vortexing, the samples were incubated at 37 °C and 250rpm shaking for 30 minutes. 350 µL of supernatant was then filtered (40 µm) into an Eppendorf containing 800 µL RPMI + 10% FBS, and the resulting filtered cells were kept on ice. The remaining tissue fragments underwent a second digestion after supplementing with 350 µL total of collagenase, DNase and RPMI at concentrations specified above per tissue type. After a second incubation at 37°C and 250rpm for 30 minutes, 350 µL of supernatant was filtered into the collection tubes containing single cells from previous filtration step. Samples were then spun at 500×g for 10 minutes, resuspended in 500 µL of Red blood cell lysis buffer (Lucigen, SS000400-D2) and incubated at room temperature for 5 minutes. 700 µL of PBS was then added, samples were spun at 500×g for 10 minutes, and then resuspended in 50 µL of PBS.
Instrument	BD FACSymphony A5
Software	Post-acquisition analyses were done using FlowJo software version 10.8
Cell population abundance	Total CD45+ cells as a proportion of live cells (Figure 4B) and total cells and total CD45+ cell counts normalized to tissue weights is shown in Figure S4B-C.
Gating strategy	Gating strategy is shown in Fig 4. Immune cell subsets were delineated from the CD45+ Zombie aqua- population and defined based on the following staining profiles: basophils (CD19-, Ly6G-, MHCII-, CD11b+, CD62L-, Ly6c+/- CD25+), dendritic cells (CD11b+, CD11C+/-, MHCII+, CD24+), eosinophils (CD19-, Ly6G-, MHCII-, CD11b-, CD62L-, Ly6clo CD25-), macrophages (CD11b+, CD11C+/-. MHCII+, CD24+/-, CD64+), mast cells (CD19-, Ly6G-, MHCII-, CD11b-, CD62L+/-, Cd11clo, SSCAlo), monocytes (CD19-, Ly6G-, MHCII-, CD11b+, CD62L+), neutrophils (CD19-, Ly6G+), natural killer cells (CD19-, Ly6G-, NK1.1+), MHCII+ other cells (CD19-, Ly6G-, CD11b+, CD11c+, MHCII+, CD24L- CD64L-), MHCII- other cells (CD19-, Ly6G-, CD11b-, CD11c+, MHCII-, CD62L+/-), B cells (CD19+, Ly6G), CD4+ T cells (CD19-, Ly6G , CD11b-, CD11c-, CD4+), regulatory T cells (Tregs) CD4+ T cells (CD19-, Ly6G , CD11b-, CD11c-, CD4+, CD25+), active CD4+ T cells (Tregs) CD4+ T cells (CD19-, Ly6G , CD11b-, CD11c-, CD4+, CD69+), naïve CD4+ T cells (CD19-, Ly6G , CD11b-, CD11c-, CD4+, CD62L+), memory CD4+ T cells (CD19-, Ly6G , CD11b-, CD11c-, CD4+, CD44+), CD8+ T cells (CD19-, Ly6G , CD11b-, CD11c-, CD8+), naïve CD8+ T cells (CD19-, Ly6G , CD11b-, CD11c-, CD8+, CD62L+), memory CD4+ T cells (CD19-, Ly6G , CD11b-, CD11c-, CD8+, CD44+).

☒ Tick this box to confirm that a figure exemplifying the gating strategy is provided in the Supplementary Information.
